# Synthesis of PtCoNiRu/C nanoparticles by spray drying combined with reduction sintering for methanol electro-oxidation†

**DOI:** 10.1039/c9ra09764c

**Published:** 2020-01-22

**Authors:** Chensiqi Yao, Hao Xu, Anjin Li, Jigang Li, Fangzhao Pang, Panchao Zhao, Jiangyun He, Wei Yi, Yunbo Jiang, Long Huang

**Affiliations:** State Key Laboratory of Advanced Technologies for Comprehensive Utilization of Platinum Metals, Kunming Institute of Precious Metals Kunming 650106 China xyz6527753@hotmail.com jyb@ipm.com.cn huanglong@ipm.com.cn

## Abstract

The controllable synthesis of carbon-supported platinum-based multicomponent alloys is important for the development and application of direct methanol fuel cells (DMFCs). In this paper, controllable synthesis of carbon-supported PtCoNiRu quaternary alloy is realized by spray drying and reduction sintering. The effects of reduction temperature on the size, morphology and catalytic properties of the metal nanoparticles were investigated. The electrochemical performance of the as-synthesized PtCoNiRu/C catalysts towards methanol electro-oxidation was studied using cyclic voltammetry (CV) and chronoamperometry. The results show that metal nanoparticles with uniform size and dispersity on the carbon surface can be obtained at a suitable sintering temperature, while the catalyst has a higher electrochemical active surface area (ECSA) and shows better catalytic activity and stability for methanol electro-oxidation. The method described in this study provides a new route for the manufacture of Pt alloy nanoparticles with higher catalytic activity and stability.

## Abstract 2

The controllable synthesis of carbon-supported platinum-based multicomponent alloys is important for the development and application of direct methanol fuel cells (DMFCs).

## Graphical abstract

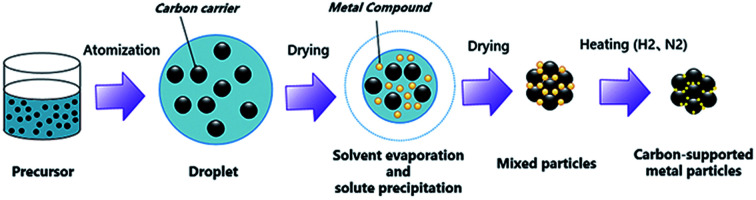

## 1. Introduction

Fuel cells, as clean and renewable energy conversion devices, have become a subject of extensive research interest due to their high power density, high energy efficiency, low operating temperature, and easy storage and transportation.^1–3^ So far, Pt-based materials are considered to be the most effective electrocatalysts for fuel cells. However, Pt is scarce and expensive, and the kinetics of small organic molecule electro-oxidation on Pt is still relatively sluggish.^4,5^ Development of Pt-based nanocatalysts with high catalytic efficiency is crucial for the large-scale application of fuel cells. Embedding Pt with other metals to form PtM bimetallic nanoparticles (NPs) has become the mainstream approach to improve electrocatalytic activity and the usage of platinum.^6–11^ Several Pt-based alloys including a second metal such as Fe, Ni, Co, Mo, Sn, Pd and Ru were explored intensively as CO-tolerant methanol oxidation reaction (MOR) electrocatalysts.^4^ According to the literature, Pt-based bimetallic alloy surfaces offer enhanced catalytic activity and stability over pure Pt due to the ‘‘bifunctional mechanism’’ and ‘‘ligand effects”.^4,12^ In addition to binary alloy catalysts, ternary, quaternary and quinary alloy catalysts have also received wide attention in recent years.^13,14^ A series of Pt–MN (M, N = Fe, Co, Ni, Ti, V, Sn, Cr, Mn, Mo, Ag, Au, Pd *etc.*) ternary alloy NPs have been synthesized and studied. Due to the synergistic effect, the activity and stability of ternary alloy and multi-metal alloy NPs may be higher than that of corresponding binary alloy NPs.^15–19^

The controllable preparation of Pt based alloy catalysts with high activity and stability by a low cost and simple method is a hindrance to the practical application of fuel cells. The spray-drying method is a well-trusted powder manufacturing method, which has been confirmed by its use in the manufacturing of dried food, fertilizers, oxide ceramics, and pharmaceuticals.^20^ The droplets, which are atomized from the precursor solution, are introduced to the solvent evaporator. Evaporation of the solvent, diffusion of solute, drying, and precipitation may occur inside the furnace to form the dry powder.^21^ There are many kinds of precursor liquids, including solute completely dissolved solution, stable system emulsion or suspension formed by solute under certain conditions (usually through emulsifier).^22^ As an effective method to prepare nanocomposite materials, spray drying has the characteristics of high degree of refinement, high operability and fine particle uniformity, which can be used to fabricate well-dispersed Pt NPs with a controllable size and morphology.^23–25^

In present study, PtCoNiRu quaternary alloy NPs were synthesized and characterized. Ni and Co alloyed with Pt to increase the electrocatalytic activity,^26^ and Ru improved the anti CO poisoning performance.^3^ PtCoNiRu quaternary alloy NPs were synthesized and evenly dispersed on the modified Vulcan XC-72 surface with high loading capacity by spray drying combined with reduction sintering. The inner structure and the electrochemical activities for MOR of the as-synthesized alloy NPs were investigated. The influences of heat treatment temperature on the microstructure and electrocatalytic performance of the PtCoNiRu/C were also discussed. The technology can be applied to the preparation of other supported alloy powders.

## 2. Experimental

### 2.1 Chemicals

All reagents and solvents were used as received without further purification. Ammonium chloroplatinate [(NH_4_)_2_PtCl_6_] and diammonium hexachlorouthenate [(NH_4_)_2_RuCl_6_] were purchased from Sino-Platinum Metals Co. Ltd (Kunming, Yunnan, P. R. China), nitrogen (N_2_, 99.99%) and hydrogen (H_2_, 99.99%) from Pengyida Co. Ltd (Kunming, Yunnan, P. R. China). Nickel chloride (NiCl_2_) was from Shanghai Darui Fine Chemicals Co., Ltd (Shanghai, P. R. China) and cobalt(ii) oxalate dehydrate (CoC_2_O_4_·H_2_O) was from Shanghai Macklin Blochemical Co., Ltd (Shanghai, P. R. China). Vulcan XC-72 was purchase from Shanghai King Chemical (Shanghai, P. R. China). 40 wt% Pt/C commercial catalysts were purchased from Johnson Matthey (Shanghai) Chemicals Ltd (Shanghai, P. R. China).

### 2.2 Modification of carbon black

A certain amount of XC-72 carbon black powder was added to acetone solution of about twice the volume of carbon powder, and the mixture was stirred for 3 h at room temperature. After that, the powder was filtered and washed several times, and then dried in N_2_ atmosphere at 50 °C for 4 h. Then the dried sample was added into a mixing solution of 10% HNO_3_ and 30% H_2_O_2_ (volume ratio HNO_3_ : H_2_O_2_ = 2 : 1) and refluxed at 60 °C for 5 hours. The sample was filtered and washed several times by deionized water to be neutral. After dried in vacuum at 50 °C, the final carbon powder was obtained after grounding.

### 2.3 Preparation of precursor suspension

The metal salts were dissolved in deionized water in a certain proportion (metal atomic ratio, Pt : Co : Ni : Ru = 3 : 1 : 1 : 1, total Pt + Co + Ni + Ru is 60 wt%), and modified carbon powder was added. After stirred for 30 minutes, the solution and carbon mixture were ultrasonically treated with an ultrasonicator operated at an ultrasonic wattage of 360 W for 1 h to ensure the uniform mixing of carbon black in water. To this, the precursor suspension was obtained.

### 2.4 Spray drying treatment

**Fig. 1:**
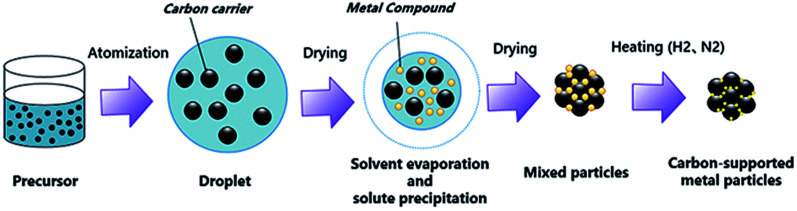
The schematic diagram of spray drying and reduction sintering process.

A commercial spray drying machine (B290, Buchi, Switzerland) was used to prepare the catalysts. A two-fluid nozzle was used as the atomizer. The temperatures at the inlet and outlet of the spray dryer were 180 °C and 130 °C, respectively. The atomization pressure was 0.6 MPa, and the feed rate of the precursor was 4 ml min^−1^. The schematic diagram of spray drying process is shown in Fig. 1.

### 2.5 Reduction sintering

The spray dried particles were sintered in H_2_/N_2_ (vol 1 : 1) at different temperatures (410 °C, 460 °C, 500 °C, respectively) followed by cooling under N_2_ atmosphere. Here, we firstly prepared PtCoNiRu/C precursors, and then sintered at 410 °C, 460 °C, 500 °C respectively in H_2_/N_2_ environment. The sample sintering at 410 °C, 460 °C and 500 °C are denoted as PtCoNiRu-410 °C, PtCoNiRu-460 °C and PtCoNiRu-500 °C, respectively. In order to determine the best sintering temperature for the activity of the quaternary alloy NPs, the powder was also sintered at 450 °C and 470 °C in the same sintering atmosphere.

### 2.6 Characterizations

The microstructures of the as-synthesized samples were observed by transmission electron microscopy (TEM) and high-resolution TEM (HRTEM) (JEOL JEM-2100) operated at 200 kV. X-ray diffraction (XRD) patterns of the synthesized powders were collected using a X-ray diffractometer (Empyrean, PANalytical, the Netherlands, CuKa radiation at 40 kV). XRD scans were recorded with a scan rate of 5° min^−1^ for 2*θ* values between 30° and 80°. Cyclic voltammograms and chronoamperometry were performed at room temperature in a standard three-electrode electrochemical cell, and measured by electrochemical workstation (Shanghai Chenhua CHI760E). The X-ray photoelectron spectroscopy (XPS) measurements were carried out on a XPS apparatus (K-Alpha^+^, Thermo fisher Scientific) with photon energy of 1486.6 eV. The composition of the catalysts was analyzed by was measured by inductively coupled plasma-atomic emission spectrometry (ICP-ASE, Optima 5300 DV, PerkinElmer, USA).

The catalyst was coated on a polished glass carbon disc (diameter 3 mm, geometric area 0.0706 cm ^2^) to form a working electrode. Ag/AgCl (saturated KCl) electrode and platinum mesh were used as the reference electrode and counter electrode, respectively, for electrochemical testing. The present study was given *versus* Ag/AgCl as a reference electrode. The electrochemical active surface area was measured in a N_2_-saturated 0.5 M H_2_SO_4_ solution at a scan rate of 0.05 V s^−1^ and the electrocatalytic activity, durability and Tafel curves for the MOR was measured in a N_2_-saturated 0.5 M H_2_SO_4_ + 0.5 M CH_3_OH solution. The electrochemical stability was tested by cyclic voltammetry for 5000 cycles from 0.2 to 1.0 V in N_2_ saturated 0.5 M H_2_SO_4_ solution.

## 3. Results and discussions

### 3.1 Structural characterizations

**Fig. 2:**
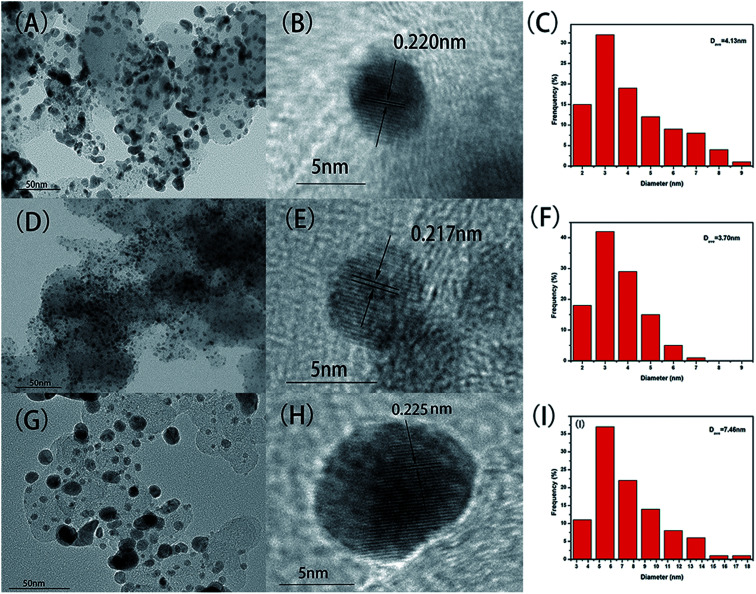
TEM images, HR-TEM images and particle size histograms of PtCoNiRu/C sintered at 410 °C (A–C), 460 °C (D–F), 500 °C (G–I), respectively.

TEM was used to characterized the distribution of as-synthesized NPs on carbon support, and the average particle size of as-synthesized NPs was calculated by statistical method. The distribution of NPs is greatly influenced by the sintering temperature as can be seen in the Fig. 2. When the sintering temperature is 460 °C (Fig. 2D), the metal particles are well dispersed and densely covered on the carbon support without obvious agglomeration. In contrast, in the case of sintering temperature of 410 °C and 500 °C (Fig. 2A and G, respectively), there are some large agglomeration deposits on the carbon support. The metal particles are nearly spherical, and size of the particles decreases first and then increases with the increase of the sintering temperature. As shown in the corresponding particle size histograms, PtCoNiRu/C-460 °C (Fig. 2F) exhibited narrow particle size distribution with an average diameter of 3.7 ± 0.9 nm. When sintered at 410 °C or 500 °C, the particles show wider size distribution with average size of 4.1 ± 1.6 nm and 7.5 ± 2.9 nm, respectively. It is clear that sintering temperature is an important factor to determine the dispersion and size uniformity of metal particles on the support. At suitable temperature, the interaction between metal ions and support materials can promote nucleation and stable growth, leading to NPs with evenly dispersion and size uniformity.

**Fig. 3:**
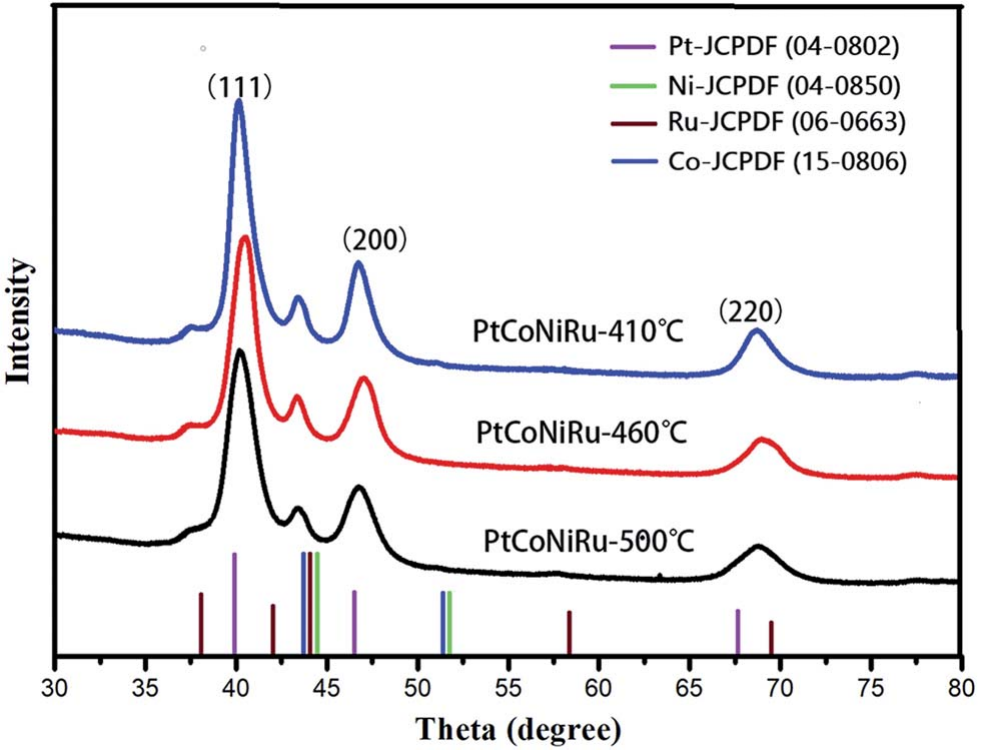
XRD scans of PtCoNiRu/C sintered at 410 °C, 460 °C, 500 °C, respectively.

HRTEM images of as-synthesized NPs are shown in Fig. 2B, E and H, it can be found that the crystal interplanar spacing of the particles first decreases and then increases with the increase of the sintering temperature, which are 0.220 nm, 0.217 nm and 0.225 nm for sintering temperature of 410 °C, 460 °C and 500 °C, respectively.^27^ The interplanar space values of all three samples are lower than that of standard Pt (111) (0.227 nm), indicating that Pt can be alloyed with Co, Ni and Ru by using the spray drying-pyrolysis method. Fig. 3 shows the XRD patterns of the as-synthesized PtCoNiRu/C powders. The XRD patterns for these powders exhibit diffraction peaks close to the (111), (200), (220) crystal planes of Pt with face-centred-cubic (fcc) phase, which confirmed the fcc structure of the as-synthesized NPs. Besides the fcc Pt-like diffraction set, no individual peak corresponding to Ru, Co or Ni was observed, which indicates the formation of Pt-based alloy NPs. Close inspection of the XRD patterns of as-synthesized powders show that 2*θ* values shift to higher values compared to Pt, showing that Co, Ni and Ru were alloyed with Pt. The sample sintering at 460 °C shows the highest 2*θ* value shifted among the three samples, indicated that in this condition the alloying degree and the amount of incorporated foreign atoms is highest, which may influence their electrochemical performance. According to the Scherrer's equation, the particle size for sintering at 410 °C, 460 °C and 500 °C is 3.3 nm, 3.6 nm and 4.0 nm, respectively, which demonstrates that the size of the metal NPs loaded on the support also increased upon sintering temperature increasing.^28,29^ These results are in good agreement with HRTEM results.

**Fig. 4:**
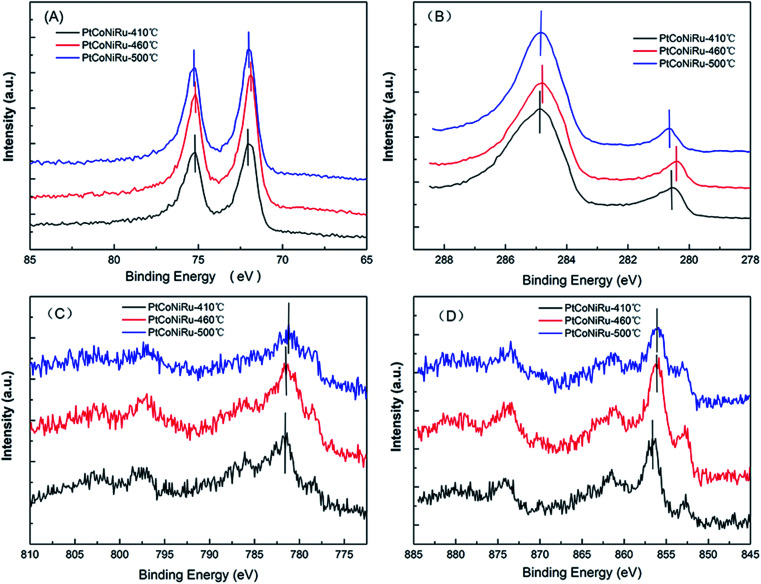
XPS spectra of Pt 4f (A), Ru 3d (B), Co 2p (C) and Ni 2p (D) of three PtCoNiRu/C sintered at 410 °C, 460 °C, 500 °C, respectively.

The XPS is a surface sensitive method for the analysis of the surface state of solid materials.^30^Fig. 4A–D show the XPS spectra of Pt 4f, Ru 3d, Co 2p and Ni 2p of the NPs, and the XPS survey spectra of three PtCoNiRu/C samples sintered at 410 °C, 460 °C and 500 °C are displayed in Fig. S1.† The binding energy values and chemical composition of the electrocatalysts are presented in Table 1, and the curve fitting of XPS peaks for Pt 4f and Ru 3d are shown in Fig. S2.† For these PtCoNiRu/C powders, binding energy of Pt is higher than that of Pt/C (4f_7/2_ = 71.0 eV; 4f_5/2_ = 74.5 eV), which is due to the electron transfer from foreign atoms (Ru, Ni and Co in this case) to Pt atoms, and clearly suggests the formation of Pt–M (M = Ru, Ni and Co) alloyed phases.^31^ As seen from Table 1, the electron binding values of other metal elements have different degree of positive shift compared with their metal elements (Ru 3d_5/2_ = 280.2 eV, Co 2p_3/2_ = 778.2 eV, Ni 2p_3/2_ = 852.6 eV), which is due to the influence of alloying. Detailed XPS analysis indicate that strong doublet peaks at ∼71.9 eV (Pt 4f_7/2_) and ∼75.2 eV (Pt 4f_5/2_) with the theoretical ratio of peak areas of 4 : 3 indicate the attribution of elemental Pt. The doublet peaks at ∼72.8 and ∼76.8 eV can be attributed to Pt^2+^ in the form of PtO or Pt(OH)_2_. Samples sintered at 410 °C, 460 °C and 500 °C are comprised of 75.1% metallic Pt and 24.9% Pt^2+^, 53.3% metallic Pt and 46.7% Pt^2+^, 53.3% metallic Pt and 46.7% Pt^2+^, respectively. Ru 3d_5/2_ peak was chosen to analyze Ru species since Ru 3d_3/2_ overlaps with C 1s peak. Samples sintered at 410 °C, 460 °C and 500 °C are comprised of 36.6% metallic Ru and 63.4% RuO_2_, 33.3% metallic Ru and 66.7% RuO_2_, 39.9% metallic Ru and 60.1% RuO_2_, respectively. The results suggest that when the sintering temperature increased from 410 °C to 460 °C, the binding energy of metal atoms decreased while more oxidized metal atoms were observed on the surface.

**Table 1:** Binding energy of Pt 4f, Ru 3d, Co 2p, Ni 2p and the Pt/Ru/Co/Ni composition ratios obtained from XPS and ICP analysis of three PtCoNiRu/C sintered at 410 °C, 460 °C, 500 °C, respectively

Binding energy (eV)	PtCoNiRu-410 °C	PtCoNiRu-460 °C	PtCoNiRu-500 °C
Pt 4f_7/2_	72.05	71.85	71.85
Pt 4f_5/2_	75.31	75.15	75.25
Ru 3d_3/2_	284.81	284.77	285.24
Ru 3d_5/2_	280.68	280.49	280.57
Co 2p_3/2_	779.30	778.93	779.55
Co 2p_1/2_	786.80	786.09	786.31
Ni 2p_3/2_	853.88	853.26	853.13
Ni 2p_1/2_	873.93	873.29	873.48
Pt : Ru : Co : Ni ratio (XPS)	1 : 0.96 : 0.02 : 4.07	1 : 0.60 : 0.56 : 2.36	1 : <0.01 : <0.01 : 1.22
Pt : Ru : Co : Ni ratio (ICP)	3 : 1.01 : 1.10 : 1.05	3 : 1.01 : 1.09 : 1.05	3 : 1 : 1.08 : 1.05

The composition of PtCoNiRu annealed at different temperatures was analyzed by ICP and XPS, as shown in Table 1. The metal atomic ratio of PtCoNiRu NPs analyzed by ICP is close to the targeted Pt : Co : Ni : Ru ratio of 3 : 1 : 1 : 1. And the Pt : Co : Ni : Ru ratio from XPS show less Pt content and more Ru content compared with the ratio obtained from ICP. Since ICP data represents the overall composition of these NPs, and XPS is a surface sensitive detection technology (1–3 nm), it is inferred that surface of the PtCoNiRu NPs may be composed of metals with approximate proportion which can be easily influenced by sintering temperature.^32,33^ The interior is mainly composed of Pt, Co and Ni, and almost all of Ru is in the surface. When sintered at 460 °C, XPS results show that the composition ratio of the four metals in the surface is the closest, compared with the results when sintered at other temperatures.

### 3.2 Electrocatalytic performance

**Fig. 5:**
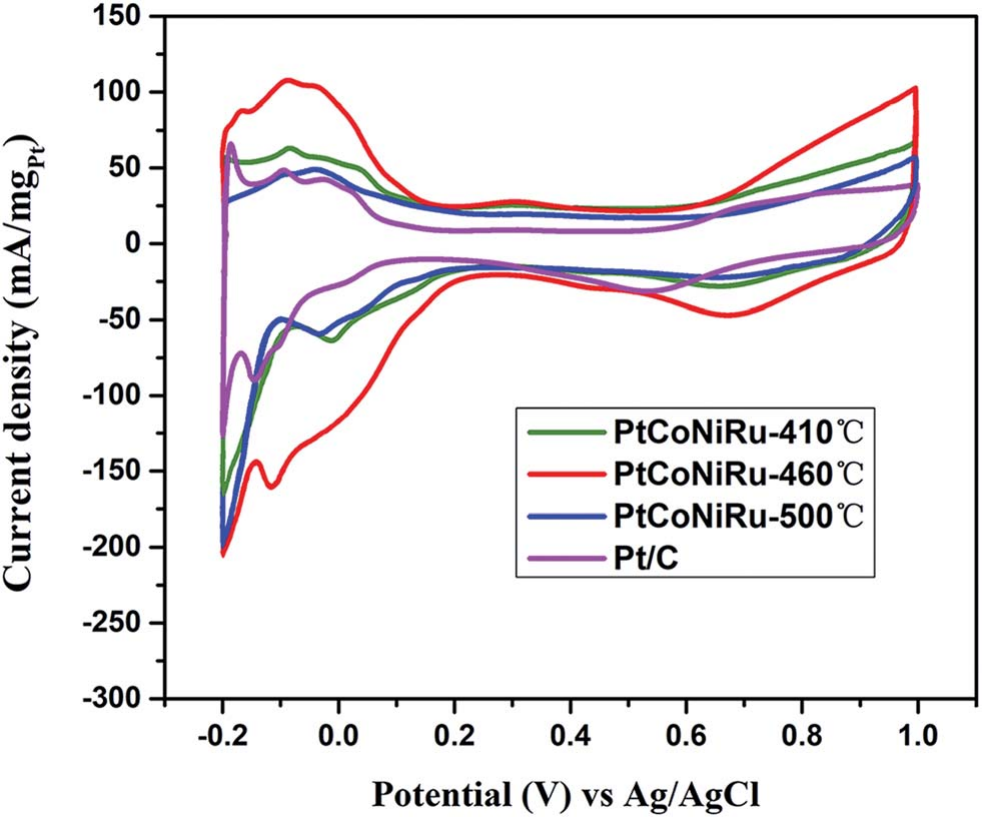
Cyclic voltammograms in N_2_-saturated 0.5 M H_2_SO_4_ solution of PtCoNiRu/C sintered at 410 °C, 460 °C, 500 °C and commercial Pt/C with the scanning rate of 0.05 V s^−1^.

Fig. 5 shows the cyclic voltammograms of commercial Pt/C and PtCoNiRu/C sintered at 410 °C, 460 °C and 500 °C in N_2_-saturated 0.5 M H_2_SO_4_ solution. The peak of hydrogen adsorption and desorption can be observed in the potential range from −0.2 to 1.0 V *vs.* Ag/AgCl in the cyclic voltammetry curves (CVs). The ECSA can be obtained by calculating the charge of hydrogen under-potential desorption (H-UPD) after correcting the charging currents of the double layer. The calculated ECSA of as-synthesized catalysts decreased with the following order: PtCoNiRu-460 °C (108 m^2^ g_Pt_^−1^) > Pt/C (68 m^2^ g_Pt_^−1^) > PtCoNiRu-410 °C (60 m^2^ g_Pt_^−1^) > PtCoNiRu-500 °C (49 m^2^ g_Pt_^−1^). The reason why PtCoNiRu-460 °C has a higher electrochemical active area is probably due to its smaller size of metal NPs and better dispersion on carbon. The low electrochemical active area of PtCoNiRu-410 °C may result from less platinum atoms on the surface. While for PtCoNiRu-500 °C, the decreased ECSA compared with PtCoNiRu-460 °C might due to their larger particles size, as illustrated form the XRD in Fig. 3.

**Fig. 6:**
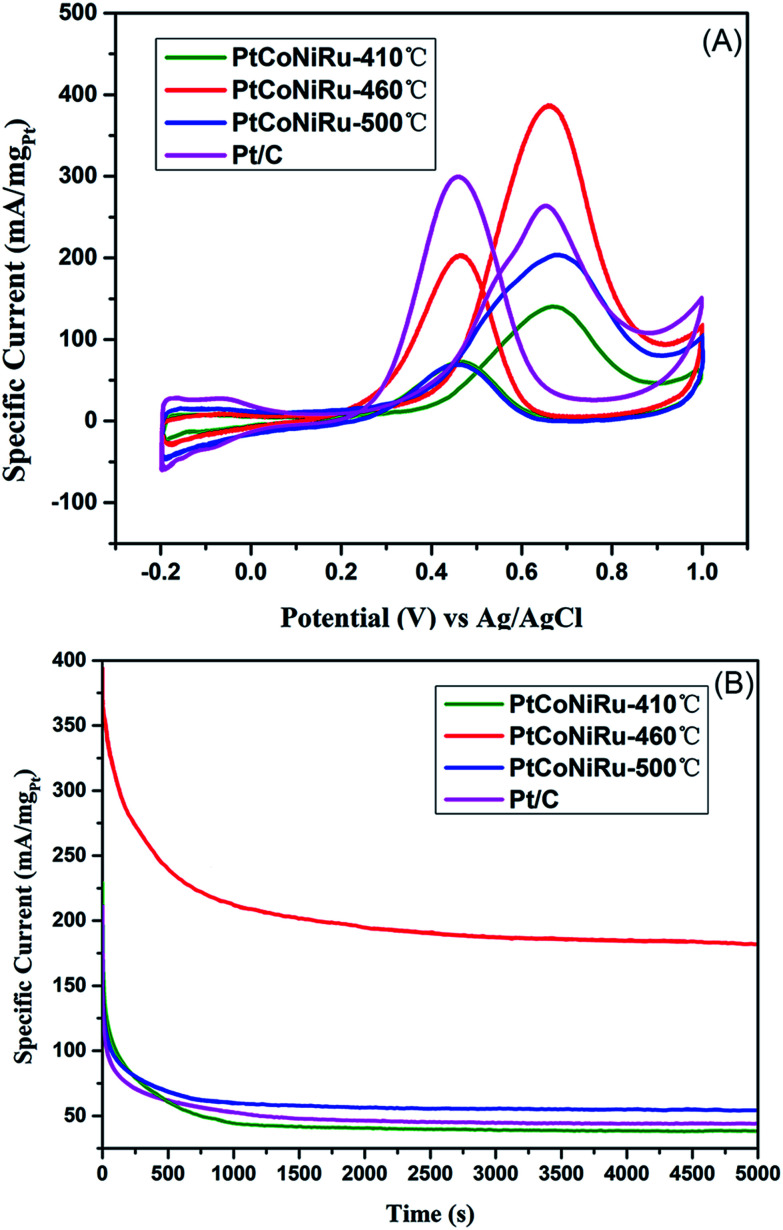
(A) Cyclic voltammograms for methanol electro-oxidation in solution containing 0.5 M H_2_SO_4_ + 0.5 M CH_3_OH on Pt_3_CoNiRu/C sintered at 410 °C, 460 °C, 500 °C and commercial Pt/C, respectively with the scanning rate of 0.05 V s ^−1^; (B) chronoamperometric curves for methanol electro-oxidation in solution containing 0.5 M H_2_SO_4_ + 0.5 M CH_3_OH on PtCoNiRu/C sintered at 410 °C, 460 °C, 500 °C and Pt/C and commercial Pt/C, respectively, at a fixed potential of 0.65 V for 1000 s.

The electrocatalytic activities of the as-synthesized PtCoNiRu/C powders towards MOR were evaluated by performing (CV) in solution containing 0.5 M H_2_SO_4_ and 0.5 M CH_3_OH. The potential range was −0.2–1.0 V with the scanning rate of 50 mV s^−1^. The voltammogram was repeated until stable and reproducible CV curve was obtained. Fig. 6A represents the CVs of MOR on three PtCoNiRu/C powders. As shown in Fig. 6A, the CVs of all the samples show a peak ascribed to methanol oxidation in forward scan and a peak related to the oxidation of carbonaceous motifs in reverse scan. It is well-known that intermediates formed during methanol oxidation such as CO and other carbonaceous species are oxidized in reverse scan. The MOR activity of the catalyst is mainly evaluated by the peak current density (*i*_p_) in the positive going scan and the onset oxidation potential (*E*_onset_). It can be seen from Fig. 6A that PtCoNiRu-460 °C and PtCoNiRu-500 °C have similar *E*_onset_ value, while PtCoNiRu-410 °C have a higher *E*_onset_ value. The lower onset potential on PtCoNiRu-460 °C and PtCoNiRu-500 °C may attribute to the bi-functional mechanism, the incorporated foreign atoms can adsorbed –OH* species at lower potential, thus facilitating the oxidation of –CO* like species. While for PtCoNiRu-410 °C, the incomplete reduction of the foreign and the reduced alloying degree makes the promotion effect less prominent.^34,35^ What's more, the surface of PtCoNiRu-410 °C sample was enriched with foreign metal while the Pt site was relatively depleted as can be seen from Table 1, which hinders the cleavage of C–H bond in the methanol molecule, thus lower the sample's ability to initial the MOR process.^36^ The *i*_p_ value of the as-synthesized sample decreased in the order of PtCoNiRu-460^o^C > Pt/C > PtCoNiRu-500 °C > PtCoNiRu-410 °C. The reason that PtCoNiRu-460 °C has the highest MOR activity was ascribed to the high dispersion and ECSA of NPs on carbon support. In addition, the functional groups on the surface of NPs are increased as the addition of other metal atoms. Meanwhile, H_2_O activation is promoted, and more OH ions and other oxygen-containing species are produced to oxidize the intermediates on the surface of active Pt. In the case of PtCoNiRu-460 °C, the synergistic effect is dominant.^37,38^ In order to optimize the sintering temperature for improving the electrocatalytic activity of the NPs. The CVs of the samples sintered at 450 °C, 460 °C and 470 °C for MOR were compared in Fig. S3,† which further proved that the quaternary alloy powder sintered at 460 °C showed the highest MOR activity. Tafel curves (Fig. S4†) have slopes of 106, 168, 118 and 120 mV per decade on PtCoNiRu-410 °C, PtCoNiRu-460 °C, PtCoNiRu-500 °C and commercial Pt/C (comm Pt/C), respectively, indicating that PtCoNiRu-460 °C significantly improved MOR kinetics.^39,40^ When the sintering temperature is low, alloying makes the effect of foreign atoms less prominent, while for the higher sintering temperature, the increased particles size and poor dispersion of NPs on the support decrease the ECSA of catalysts, thus lowering the mass activity. The sintering temperature of 460 °C balances the opposite factors and shows highest MOR activity in the studied condition.

**Fig. 7:**
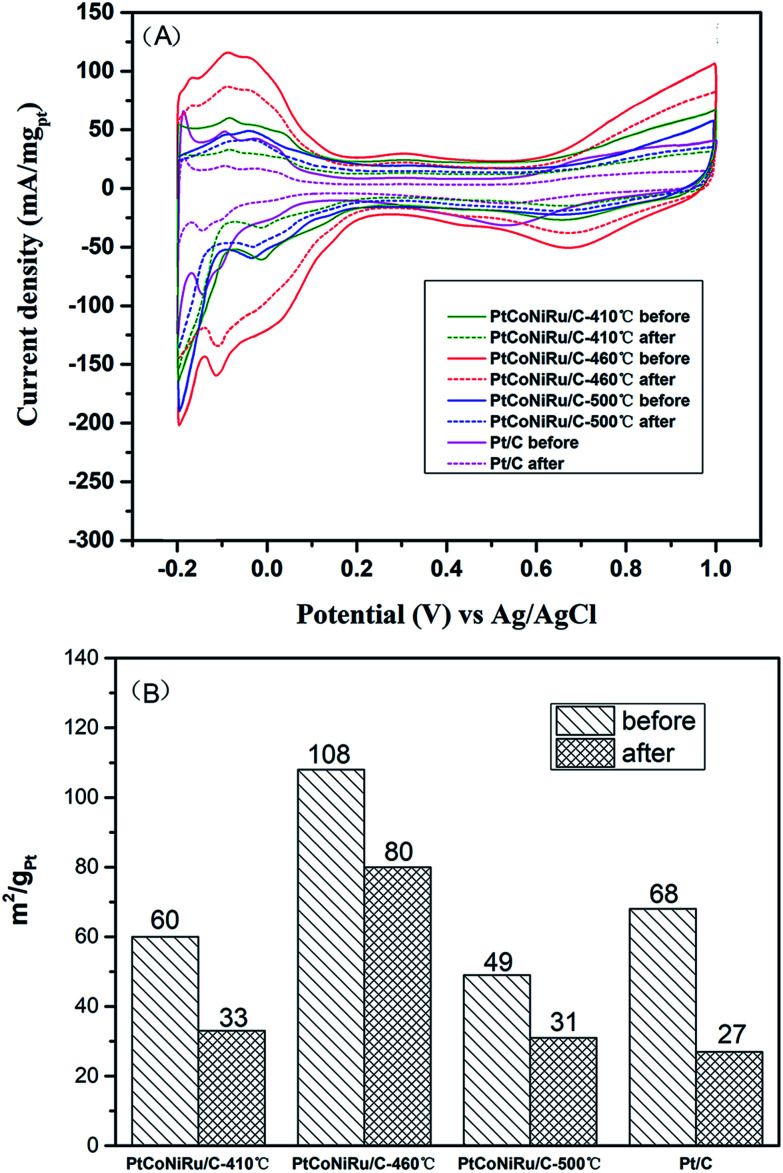
(A) Cyclic voltammograms curves of PtCoNiRu/C sintered at 410 °C, 460 °C, 500 °C and commercial Pt/C before and after 5000 cycles in N_2_-saturated 0.5 M H_2_SO_4_ solution. (B) ECSA of PtCoNiRu/C sintered at 410 °C, 460 °C, 500 °C and commercial Pt/C before and after 5000 cycles in N_2_-saturated 0.5 M H_2_SO_4_ solution.

The MOR durability of the samples was also tested by performing chromoamperometry experiments at 0.65 V in 0.5 M H_2_SO_4_ containing 0.5 M CH_3_OH. Fig. 6B shows the current–time (*I*–*t*) curves of methanol oxidation on four catalysts. As can be seen from the figure, the catalyst sintered at 460 °C shows the highest initial current density and maintains the highest current density during the whole time range. The currents of all samples decrease gradually under the high potential and reach a stable state after 300 s of operation. The initial decay in current density observed in all the samples can be attributed to the formation of CO-like species formed on the electrode surfaces during methanol electro-oxidation. Based on this current at 300 s, the decay rates of all alloy samples is lower than that of Pt/C, which is ascribed to the presence of Ru, which can change the adsorption state of CO and reduce the CO coverage on the active site.^41^ The highest residual current of PtCoNiRu/C-460 °C demonstrates the NPs treated at 460 °C has the best catalytic durability. We tentatively ascribed the enhanced durability of PtCoNiRu/C-460 °C over the other two catalysts to the improved alloying effect. The alloying of Ru, Ni, and Co atoms into Pt lattice can facilitate the removal of CO-like species generated during MOR processes, thus free the Pt-site from CO-like poisoning species and available for MOR.^42,43^ The electrochemical stability of these samples was further investigated in N_2_-saturated 0.5 M H_2_SO_4_ solution by CV scanning from −0.2 to 1.0 V for 5000 cycles. Fig. 7A compares the CVs of these catalysts before and after 5000 voltammetric cycles, and ECSA for these catalysts were caculated and plotted in Fig. 7B. After 5000 cycles, the ECSA losses of the PtCoNiRu/C-410 °C, PtCoNiRu/C-460 °C, PtCoNiRu/C-500 °C and commercial Pt/C are 45%, 26%, 37% and 60%, respectively. It can be concluded that the alloying state of the PtCoNiRu-460 °C NPs is the most stable, and the particles are not easy to grow and agglomerate during scanning process, because the ESCA depends on the particle size and alloy degree to some extent. The significantly enhanced electrochemical catalytic stability of PtCoNiRu- 460 °C is most likely consistent with structural stability results from the best solid solubility and alloying degree when the sintering temperature is 460 °C. The increase of surface segregation and alloying degree of Pt and Ru (according the XPS results) can also prevent the dissolution of Ni and Co in the alloy surface.^37^ While the interaction between NPs and carbon support surface may also play an important role in the electrocatalytic activity and stability of NPs.

## 4. Conclusions

In summary, controllable synthesis of PtCoNiRu/C catalyst can be achieved by spray drying and reduction sintering. It can be found that the microstructure and electrochemical properties of the catalyst are very sensitive to the sintering temperature. The spray-dried powder sintered at a suitable temperature shows good dispersion, small and uniform size, and excellent electrocatalytic ability for MOR. The PtCoNiRu-460 °C catalysts exhibit significantly higher activity for MOR as compared commercial Pt/C electrocatalyst. The chronoamperometry results measured at 0.65 V (*vs.* Ag/AgCl) after 5000 s exhibits a current density higher than commercial Pt/C under identical conditions. Compared with commercial catalysts, the catalysts prepared in this study show higher catalytic activity and excellent stability in terms of electrochemical performance with simple preparation method and short production cycle, which proves that the catalyst is promising to be used as fuel cell catalysts. In perspective, the approach can be extended to be a new micro-preparation technology for the preparation of other carbon-supported multi-component alloy materials.

## Conflicts of interest

There are no conflicts to declare.

## Supplementary Material

RA-010-C9RA09764C-s001
