# Supplementary material for: Synthesis of PtCoNiRu/C nanoparticles by spray drying combined with reduction sintering for methanol electro-oxidation
Source: RSC Adv. 2020 Jan 22;10(6):3579–87. doi: 10.1039/c9ra09764c (PMC9048833; doi:10.1039/c9ra09764c)
Supplement: RA-010-C9RA09764C-s001 [file RA-010-C9RA09764C-s001.pdf]

Electronic Supplementary Material (ESI) for RSC Advances.
This journal is © The Royal Society of Chemistry 2020

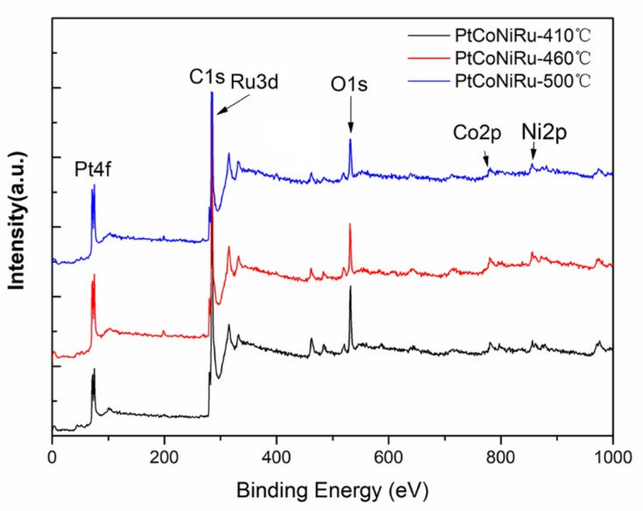

Figure S1. The XPS survey spectra of three PtCoNiRu/C samples sintered at 410 °C, 460 °C and 500 °C

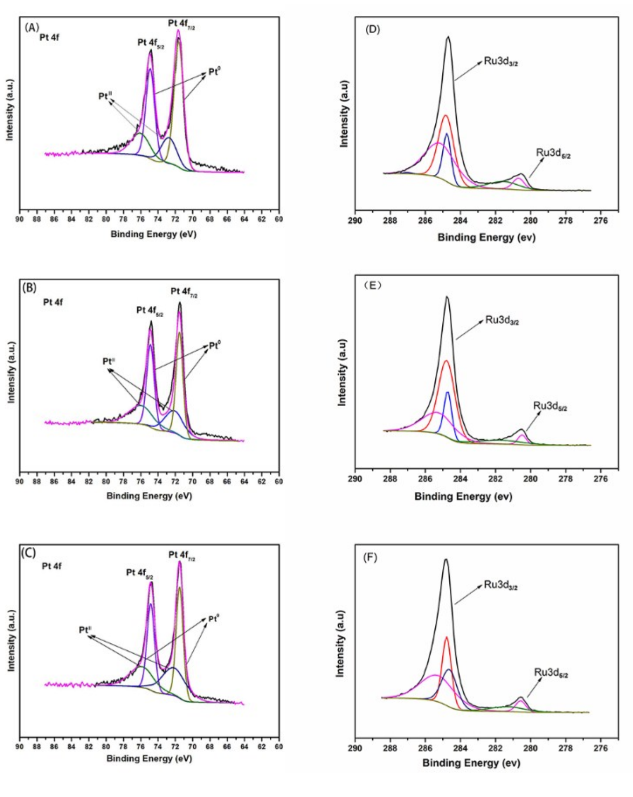

Figure S2. Curve fiting of XPS spectra for Pt4f (A), (B), (C) and Ru3d (D),(E),(F) of three PtCoNiRu/C sintered at 410 °C, 460 °C, 500 °C, respectively

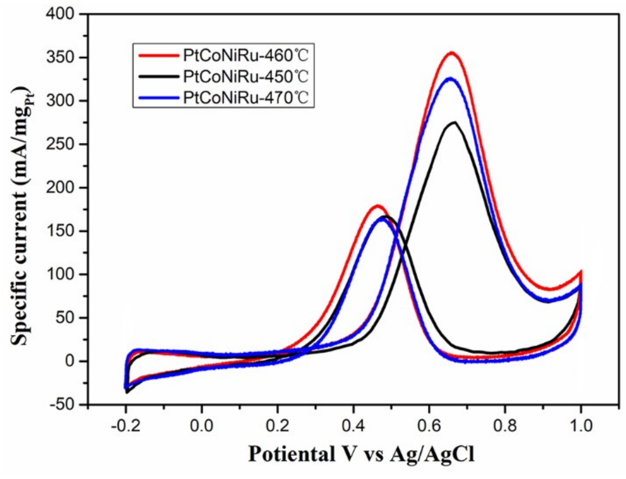

Figure S3. Cyclic voltammograms for methanol electro-oxidation in solution containing 0.5 M H_2SO_4 + 0.5M CH_3OH on PtCoNiRu/C sintered at 450 ^oC, 460 ^oC, 470 ^oC, respectively with the scanning rate of 0.05 V s^{-1}

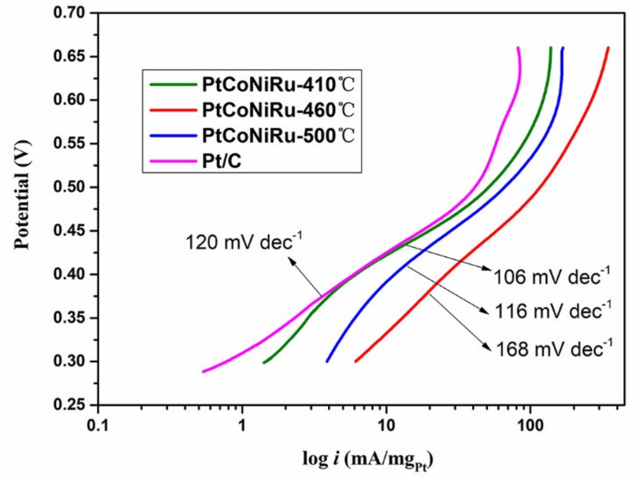

Figure S3. Cyclic voltammograms for methanol electro-oxidation in solution containing 0.5 M H_2SO_4 + 0.5M CH_3OH on PtCoNiRu/C sintered at 450 ^oC, 460 ^oC, 470 ^oC, respectively with the scanning rate of 0.05 V s^{-1}
